# Personalized probiotic strategy considering bowel habits: impacts on gut microbiota composition and alleviation of gastrointestinal symptoms via Consti-Biome and Sensi-Biome

**DOI:** 10.3389/fnut.2024.1302093

**Published:** 2024-02-16

**Authors:** Uigi Min, Yoo-Jeong Jin, You Jin Jang, Jonghyun Lim, Byung-Yong Kim

**Affiliations:** R&D Center, Chong Kun Dang Healthcare, Seoul, Republic of Korea

**Keywords:** gut microbiome, probiotics, personalized probiotics, functional gastrointestinal disorder, constipation, diarrhea, microbiome diversity, stool consistency

## Abstract

Personalized probiotic regimens, taking into account individual characteristics such as stool patterns, have the potential to alleviate gastrointestinal disorders and improve gut health while avoiding the variability exhibited among individuals by conventional probiotics. This study aimed to explore the efficacy of personalized probiotic interventions in managing distinct stool patterns (constipation and diarrhea) by investigating their impact on the gut microbiome and gastrointestinal symptoms using a prospective, randomized, double-blind, placebo-controlled clinical trial design. This research leverages the multi-strain probiotic formulas, Consti-Biome and Sensi-Biome, which have previously demonstrated efficacy in alleviating constipation and diarrhea symptoms, respectively. Improvement in clinical symptoms improvement and compositional changes in the gut microbiome were analyzed in participants with predominant constipation or diarrhea symptoms. Results indicate that tailored probiotics could improve constipation and diarrhea by promoting *Erysipelotrichaceae* and *Lactobacillaceae*, producers of short-chain fatty acids, and regulating inflammation and pain-associated taxa. These findings suggest the potential of tailored probiotic prescriptions and emphasize the need for personalized therapeutic approaches for digestive disorders.

**Clinical trial registration**: https://cris.nih.go.kr/cris/index/index.do, identifier KCT0009111.

## Introduction

Advances in sequencing technologies have enabled exploration of the human gut microbiome, which is a reservoir of diverse microorganisms crucial for overall health (1). Extensive projects such as the Human Microbiome Project (HMP) and Metagenomics of the Human Intestinal Tract (MetaHIT) have highlighted the profound influence of microbiome diversity and compositional clusters on various aspects of human physiology (2, 3). Notably, recent elucidation of the mechanisms through which the gut microbiome directly regulates gut motility underscores its pivotal role in health maintenance (4).

Functional gastrointestinal disorders encompassing constipation and diarrhea collectively affect approximately 40% of the global population, significantly impacting their quality of life (5). Constipation is characterized by hard stools or associated straining, whereas diarrhea manifests as loose stools and abdominal pain, potentially leading to dehydration, malabsorption, and electrolyte imbalance (6). Despite these disparate symptoms, gut dysbiosis is a common factor (7).

Probiotics, defined as “live microorganisms which when administered in adequate amounts confer a health benefit on the host” have traditionally been employed to manage gastrointestinal disorders, including constipation and diarrhea (8, 9). By restoring gut microbiome balance and reinforcing intestinal barrier function, probiotics offer a promising avenue to alleviate symptoms and enhance gut health (10).

However, probiotic efficacy exhibits interindividual variability. Responses to probiotic interventions differ and are influenced by variations in gut microbiome diversity, microbial composition, and metabolic profiles (11). To address this variability, the concept of “personalized probiotics” has emerged, where probiotic interventions are tailored to individual gut microbiome characteristics (12). Different gut microbiome profiles are associated with ethnicity, age, diet, lifestyle, and stool consistency (13–15). Notably, stool consistency, a primary indicator distinguishing constipation from diarrhea, significantly impacts gut microbiome composition and diversity (13, 16). Prior research on irritable bowel syndrome (IBS) emphasized the need for distinct therapeutic strategies based on stool patterns (constipation or diarrhea) (7). However, attempts to develop probiotic prescriptions that address bowel symptoms remain limited.

This study aimed to explore the potential of personalized probiotics as effective interventions for managing distinct stool patterns (constipation and diarrhea) by investigating their impact on gut microbiome states and clinical symptoms. By focusing on the influence of gut microbes on bowel habits, we examined the practicality of tailored probiotics through stool microbiome analysis and a clinical symptom assessment.

Ultimately, this study aimed to elucidate the interplay between the gut microbiome and gastrointestinal health and contribute to the development of efficacious therapeutic strategies for gastrointestinal disorders through personalized probiotic prescriptions.

## Materials and methods

### Participant recruitment

The research protocol underwent ethical review and approval by the Korea National Institute for Bioethics Policy (KoNIBP) (IRB: P01-202209-06-001) before participant recruitment. This study was registered in the Korean Clinical Trial Registry (CRIS; trial number KCT0009111). Prior to enrolment, all participants received an explanation of the study plan and provided voluntary informed consent, including the right to withdraw from the study without prejudice. Data confidentiality and security were ensured via regular monitoring throughout the study.

### Inclusion and exclusion criteria

Adults aged 19–75 years with bowel irregularities were recruited from the website of Chong Kun Dang Healthcare. Participants were categorized based on the major symptoms of functional constipation and diarrhea according to the ROME IV criteria (6). The Insensitive Gut (IG) group consisted of individuals with a Bristol Stool Score (BSS) ≤2, bowel movements ≤2 times per week, excessive straining during defecation, or a sense of incomplete evacuation. The Sensitive Gut (SG) group included individuals with a BSS ≥5, bowel movements ≥2 times daily, or discomfort during defecation. The exclusion criteria encompassed pregnant, breastfeeding, or intending-to-conceive women; individuals using dietary supplements or medications affecting gastrointestinal function; and those who underwent gastrointestinal surgery.

### Study design

This study employed a prospective, randomized, double-blind, placebo-controlled clinical trial design to investigate the efficacy of personalized probiotic prescriptions for constipation and diarrhea-related stool patterns. Remote assessments were conducted at the baseline, intervention period, and endpoint at the R&D Center of Chong Kun Dang Healthcare. Participants completed online questionnaires through a link provided for remote screening. The screening phase assessed the eligibility and exclusion criteria, and baseline and endpoint fecal samples were collected for microbiome analysis. A two-week washout period preceded probiotic intervention, and probiotic and prebiotic consumption was prohibited during the intervention period. The wash-out period was enforced to eliminate any potential influence on the composition of gut microbiome from routine probiotic intake. The intervention lasted for four weeks, and compliance was monitored through daily intake logs. Participants with insensitive and sensitive bowels were randomly assigned to the test or placebo group using a blocked randomization method, with blinding maintained throughout the study.

### Probiotic intervention

The IG and SG groups received personalized probiotic interventions: Consti-Biome and Sensi-Biome, respectively. Consti-Biome was formulated with six probiotic strains, including SynBalance® SmilinGut (*Lactiplantibacillus plantarum* PBS067, *Lacticaseibacillus rhamnosus* LRH020 and *Bifidobacterium animalis* subsp. *lactis* BL050; Roelmi HPC), *L. plantarum* UALp-05 (Chr. Hansen), *Lactobacillus acidophilus* DDS-1 (Chr. Hansen), and *Streptococcus thermophilus* CKDB027 (Chong Kun Dang Bio). Sensi-Biome also comprised six probiotic species, including *B. lactis* UABla-12 (Chr. Hansen), *B. bifidum* BB-06 (Danisco), *L. acidophilus* DDS-1 (Chr. Hansen), *L. plantarum* UALp-05, *S. thermophilus* CKDB027, and *Lactococcus lactis* MG5125 (Mediogen). The probiotic and placebo capsules were manufactured and packaged by Seoheung Healthcare Co., Ltd. (Cheong-Ju, Korea). Probiotic capsules were formulated to provide a daily dose of 1 × 10^10^ colony-forming units (CFU) per capsule (potency of not less than 1 × 10^10^ CFU/day). The placebo consisted of maltodextrin and contained the same amount of excipients (corn starch and rice powder) as the probiotic capsules. The probiotic and placebo capsules were visually and structurally indistinguishable.

### Questionnaires and assessment metrics

The BSS and stool frequency were used to assess improvements in bowel habits. The BSS employed a 7-point scale of illustrations from which participants could choose, whereas stool frequency required participants to select one of four options: 1 (less than three times a week), 2 (3–4 times a week), 3 (five times a week to once daily), and 4 (twice daily or more). Additionally, the amelioration of gut symptoms was evaluated by assessing incomplete evacuation, straining, urgency, abdominal discomfort, and abdominal pain. Each gut symptom questionnaire was rated on a 5-point Likert scale.

### Sample collection and microbiome analysis

Fecal samples were collected using fecal collection kits (NBG-1C; NobleBio Inc., Hwaseong, Korea) containing a preservative buffer for microbiome analysis. The samples were shipped to the laboratory and stored at −80°C until further processing. Genomic DNA was extracted from the thawed samples using the Omega Mag-Bind DNA Prep Kit (Omega Bio-tek Inc., Norcross, GA, United States), and the V4 region of the bacterial 16S rRNA gene amplified using the 515F–806R primer pair (17). PCR products were sequenced on an Illumina i-Seq 100 system (Illumina, Inc., San Diego, CA, United States) at the R&D Center of the Chong Kun Dang Healthcare (Seoul, Korea). Sequence data were subjected to clustering of OTU representative sequences at 98% using a pipeline generated in the CLC Genomics Workbench 22.0 (QIAGEN, Aarhus, Denmark) at the R&D Center of the Chong Kun Dang Healthcare. Taxonomic richness was calculated using the SILVA 115 database (18) as the reference database. Differential abundance analysis (DAA) was performed using an embedded tool in the CLC Genomics Workbench. Rarefaction depth was determined based on the minimum read count per sample, and alpha diversity indices computed using the “microbiome” package in the R statistical software [v3.1.0; (19)]. Beta diversity indices were calculated using the “vegan” package, whereas Spearman’s correlation and scatter plot analyses executed and visualized using the “ggplot2” package. For post-intervention microbiome marker exploration, linear discriminant analysis effect size (LEfSe) analysis was conducted using the “MicrobiomeMarker” package.

### Statistical analysis

Wilcoxon signed-rank tests were used to compare bowel habit assessments, alpha diversity indices, and relative abundances of the gut microbiota within the groups before and after intervention. Wilcoxon rank-sum tests with Bonferroni correction were used to compare post-intervention characteristics between the test and placebo groups. PERMANOVA was used to compare beta diversity. Statistical analyses were conducted using R statistical software in the RStudio environment, with *p*-values <0.05 considered statistically significant.

## Results

### Participant recruitment and baseline characteristics

**Figure 1:**
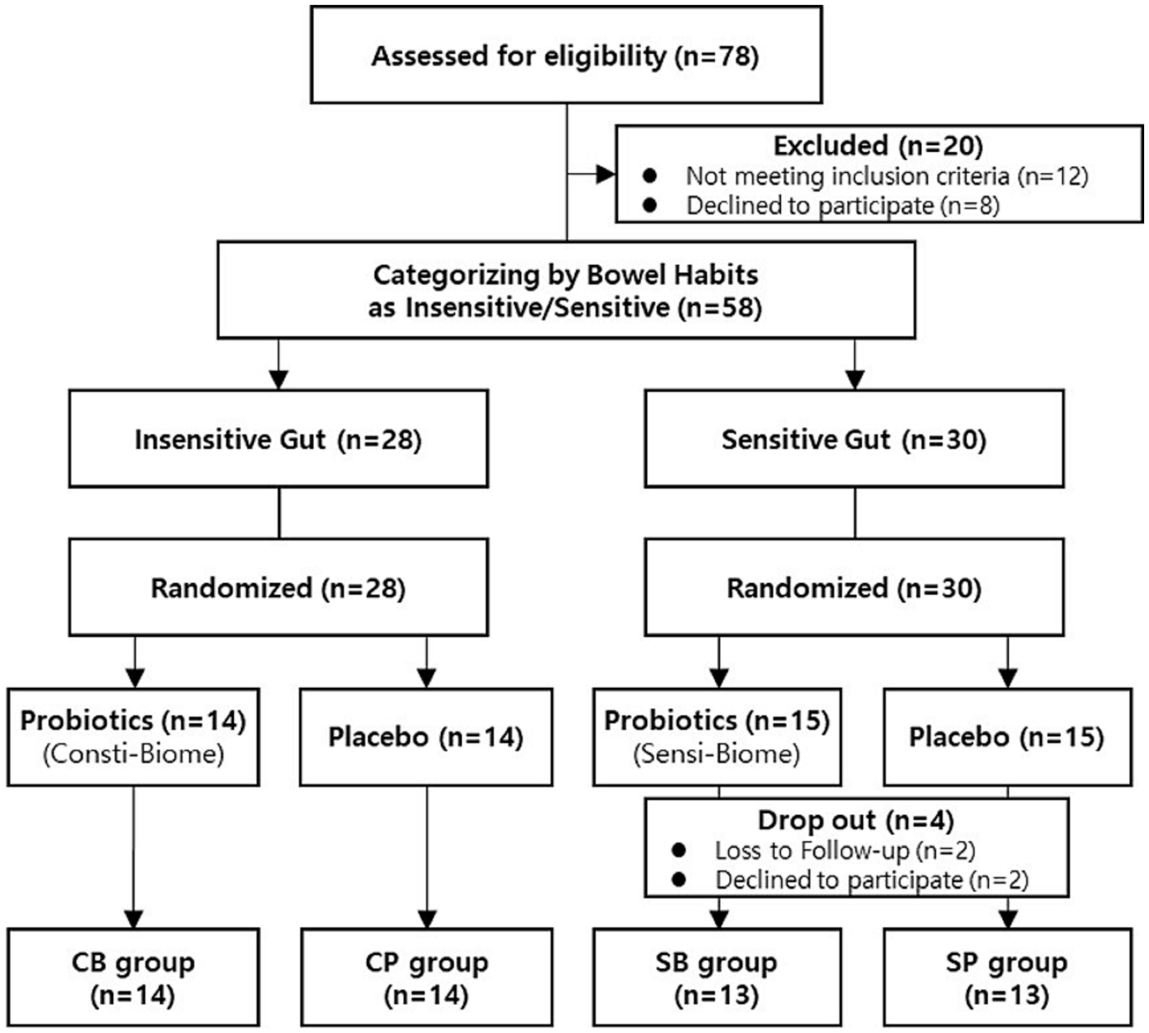
Participant flow chart.

A total of 78 participants aged between 19–75 years who self-reported experiencing bowel movement issues were recruited from September–November 2022. Among the recruited participants, 20 were excluded from the intervention because they did not meet the inclusion criteria or had met the exclusion criteria, such as reporting no disorders or being older. A total of 58 participants successfully passed the screening process and were categorized into the IG (*n* = 28) and SG groups (*n* = 30) based on their bowel habits. Through block-randomized allocation, the IG group comprised 14 participants in the intervention group and 14 in the placebo group, whereas the SG group consisted of 15 participants in both the intervention and placebo groups. During the intervention, all participants in the IG group completed the consumption, whereas four participants in the SG group, dropped out because of voluntary withdrawal and compliance failure (Figure 1). There were no statistically significant differences in clinical characteristics, baseline symptoms, age, or sex between the intervention and placebo groups in either of the IG and SG groups (Table 1).

**Table 1:** Baseline characteristics of participants in the Insensitive Gut (*n* = 28) and Sensitive Gut (*n* = 26) groups.

Group		Insensitive Gut (*n* = 28)		*p-*value	Sensitive Gut (*n* = 26)		*p*-value
		Probiotics (*n* = 14)	Placebo (*n* = 14)	–	Probiotics (*n* = 13)	Placebo (*n* = 13)	–
Sex (Male/Female)		6/8	6/8	–	4/9	7/6	–
Average age ± SD (Range)		43.7 ± 12.8 (26–65)	44.5 ± 8.5 (34–63)	0.86	46.6 ± 11.4 (29–71)	43.4 ± 9.6 (24–63)	0.60
Gut symptoms ± SD	Stool consistency (BSS)	2.7 ± 0.9	2.6 ± 0.9	0.84	4.5 ± 1.2	4.7 ± 1.2	0.75
	Stool frequency	2.8 ± 0.9	2.6 ± 0.6	0.63	3.2 ± 0.4	3.0 ± 0.6	0.43
	Incomplete evacuation	1.9 ± 1.2	2.1 ± 1.3	0.77	2.2 ± 0.9	1.9 ± 0.6	0.47
	Straining	2.4 ± 1.0	2.5 ± 1.1	0.74	2.2 ± 1.0	1.9 ± 0.6	0.49
	Urgency	1.9 ± 0.8	2.3 ± 0.9	0.29	2.2 ± 1.0	2.2 ± 0.9	0.85
	Abdominal Discomfort	1.9 ± 0.8	2.0 ± 1.1	0.72	1.5 ± 0.8	1.7 ± 0.8	0.64
	Abdominal pain	1.4 ± 0.6	1.6 ± 0.8	0.46	1.2 ± 0.4	1.4 ± 0.5	0.42

The *p*-values indicate statistical significance between the intervention and placebo groups within each gut group.

### Bowel habit improvement

**Figure 2:**
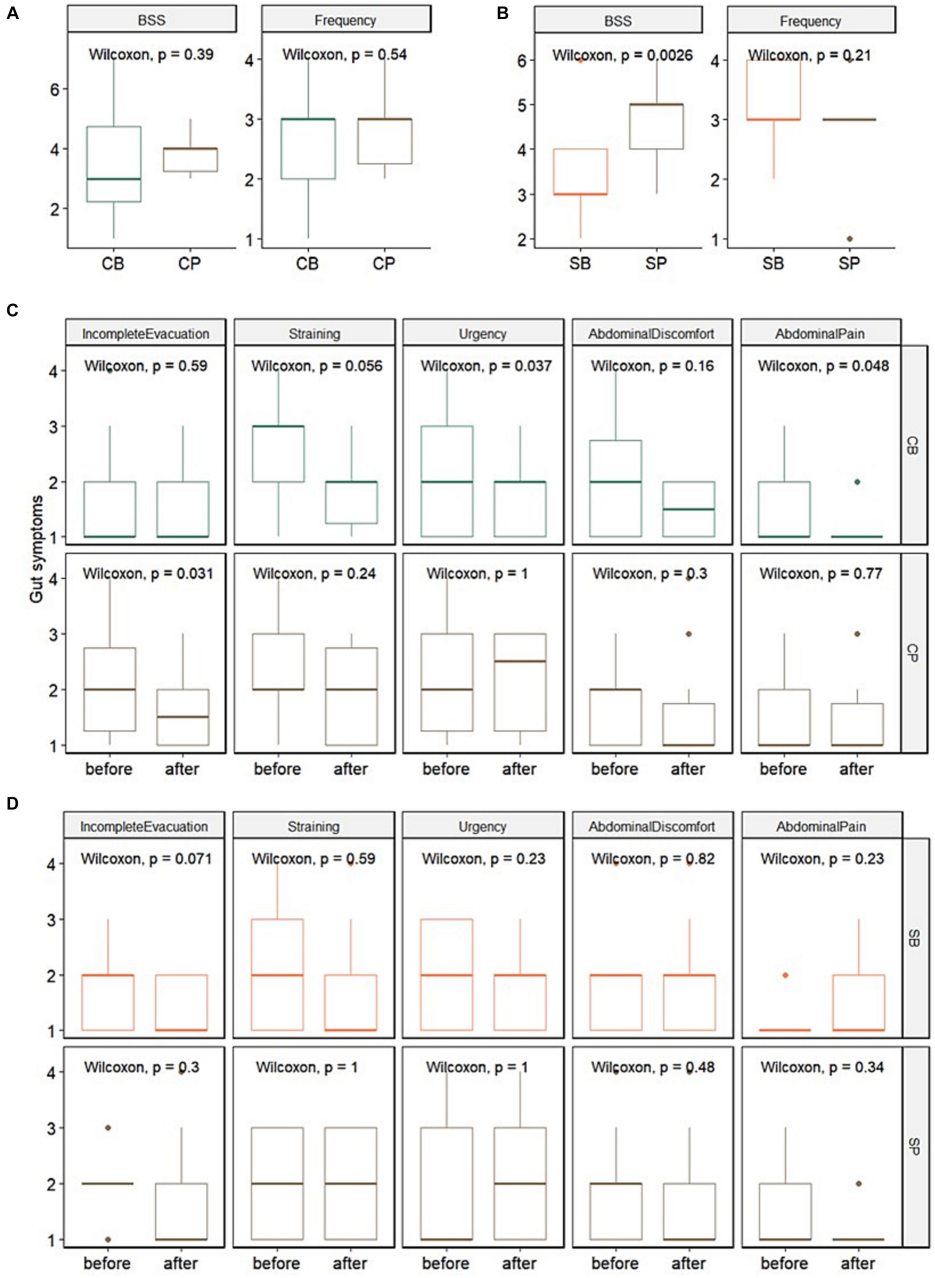
Impact of Consti-Biome (CB; *n* = 14) and Sensi-Biome (SB; *n* = 13) on gastrointestinal symptoms in the Insensitive Gut and Sensitive Gut groups compared with that in each placebo group (CP and SP; *n* = 14 and *n* = 13, respectively). Stool consistency and frequency measurements **(A,B)** and evaluations of other gastrointestinal symptoms **(C,D)** during the 4-week intervention period. These were analyzed using Wilcoxon rank-sum and signed-rank tests.

In the IG group, no significant differences in stool consistency (assessed using the BSS) and frequency were observed between the probiotics (CB) (3.4 ± 1.7 and 2.6 ± 1.1, respectively) and placebo (CP) groups (3.8 ± 0.6 and 2.9 ± 0.7, respectively) at the 4-week endpoint. Among the gut symptoms, there were no significant differences in reduction of straining and abdominal pain between the CB (1.9 ± 0.7 and 1.2 ± 0.4, respectively) and CP groups (1.9 ± 0.8 and 1.5 ± 0.9, respectively). However, a trend of reduced straining (*p* = 0.056) and a significant decrease in urgency (*p* = 0.037) and abdominal pain (*p* = 0.048) were observed in the CB group. In contrast, no significant changes in straining (*p* = 0.24), urgency (*p* = 1), or abdominal pain (*p* = 0.77) were observed in the CP group (Figures 2A,C).

For the SG group, at the 4-week endpoint, stool consistency significantly improved in the probiotics (SB) group (3.2 ± 1.1) compared with that in the placebo (SP) group (4.7 ± 0.9), whereas stool frequency showed no significant change. Although no significant differences were observed in gut symptoms after four weeks, a trend of reduced incomplete evacuation (*p* = 0.071) was evident in the SB group (Figures 2B,D).

### Microbiome modulation

Alpha diversity analyses, including Shannon, Chao1, inverse Simpson, and observed OTU indices, were performed on both the probiotics and placebo groups within the IG and SG groups at the 4-week endpoint or before and after intervention within each group. No significant differences in alpha diversity were observed between the groups (Supplementary Table S1). Beta diversity analysis, based on the Bray–Curtis dissimilarity using PCoA, and PERMANOVA, showed that there were no significant differences in beta diversity between the probiotics and placebo groups at the 4-week endpoint or before and after intervention within each group (Supplementary Figure S1A).

To investigate the role of personalized microbiome modulation in the IG and SG groups, DAA of specific gut microbial taxa at the family level was performed. In the IG group, the following taxa differed significantly: *Succinivibrionaceae* (*p* < 0.01), *Micrococcaceae* (*p* < 0.01), *Porphyromonadaceae* (*p* = 0.01), *Prevotellaceae* (*p* = 0.01), *Peptococcaceae* (*p* = 0.03), and *Alcaligenaceae* (*p* = 0.03). Characteristic taxa in the SG group were *Enterococcaceae* (*p* < 0.01), *Planococcaceae* (*p* < 0.01), *Enterobacteriaceae* (*p* < 0.01), *Nocardiaceae* (*p* < 0.01), and *Staphylococcaceae* (*p* = 0.02) (Table 2).

**Table 2:** Characteristic microbial taxa at the family level for the Insensitive Gut (*n* = 28) and Sensitive Gut (*n* = 26) groups at baseline.

Taxon name	Log₂ fold change Insensitive Gut *vs* Sensitive Gut (>0: Enriched in Insensitive Gut, <0: Enriched in Sensitive Gut)	*p-value*
*Succinivibrionaceae*	9.42	<0.01
*Enterococcaceae*	−7.17	<0.01
*Planococcaceae*	−6.28	<0.01
*Enterobacteriaceae*	−3.13	<0.01
*Micrococcaceae*	2.97	<0.01
*Nocardiaceae*	−3.20	<0.01
*Porphyromonadaceae*	1.42	0.01
*Prevotellaceae*	2.37	0.01
*Staphylococcaceae*	−1.87	0.02
*Peptococcaceae*	1.71	0.03
*Alcaligenaceae*	1.04	0.03

The *p*-values indicate statistical significance for differential abundance analysis.

**Figure 3:**
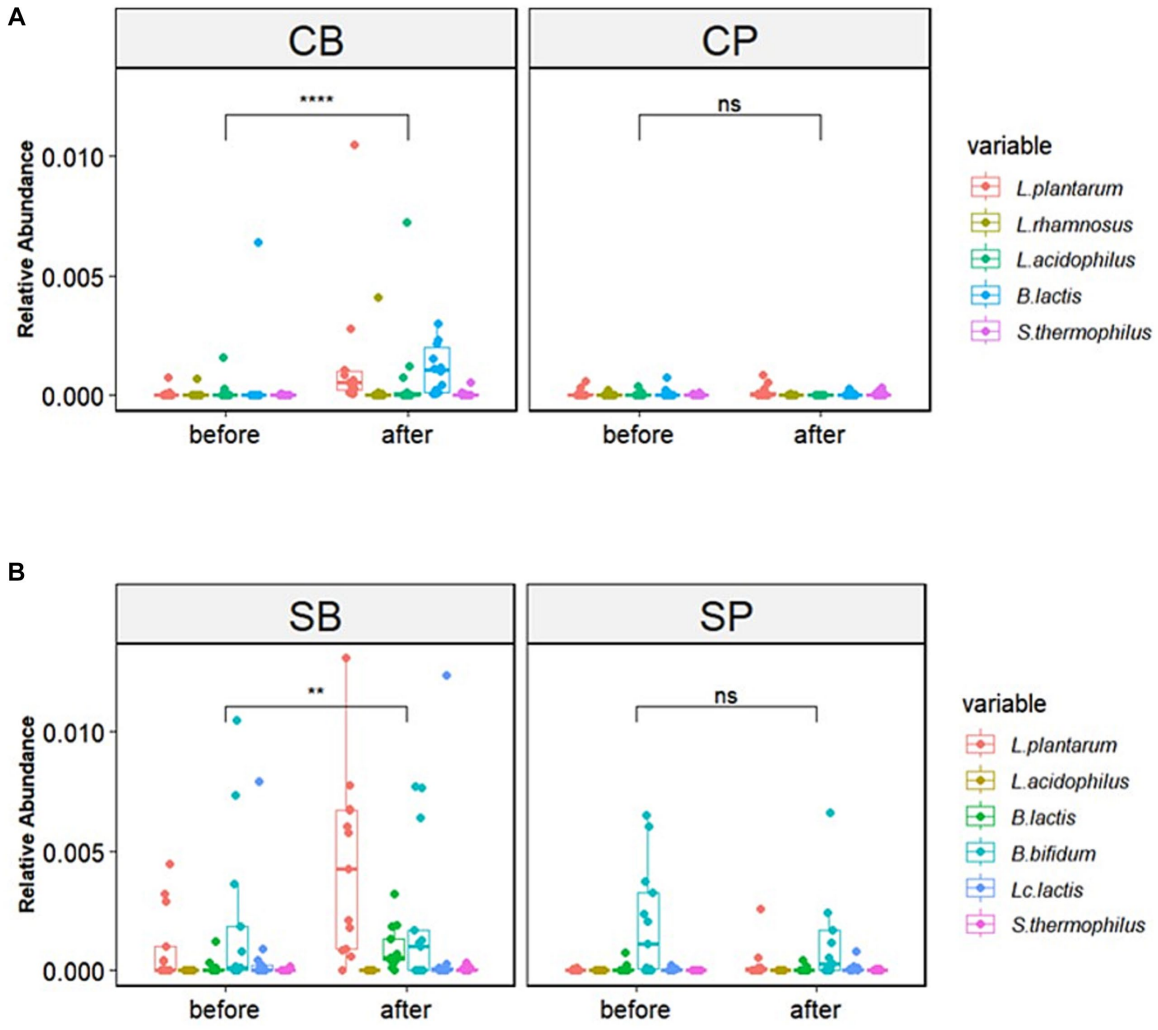
Relative abundance of probiotic strains in the Consti-Biome (CB; *n* = 14) and Sensi-Biome (SB; *n* = 13) groups before and after intervention, compared with that of the respective placebo groups (CP and SP; *n* = 14 and *n* = 13, respectively). Relative abundances in the **(A)** CB and CP, **(B)** SB and SP groups. Consti-Biome and Sensi-Biome consist of six strains each, represented as five strains in the figure owing to Consti-Biome containing two strains with identical species (*Lactiplantibacillus plantarum*). Wilcoxon signed-rank tests were performed for each comparison, and significance levels denoted with the following symbols: ns (not significant), ** (*p* < 0.01), and **** (*p* < 0.0001). Additionally, comparisons between the CB and SB groups and the corresponding placebo groups (CP and SP) after intervention also showed significant differences (data not shown).

The six strains of probiotics in Consti-Biome and Sensi-Biome significantly increased in relative abundance after intervention in the probiotics groups (CB and SB) groups, whereas no significant changes were observed in the placebo groups (CP and SP). Moreover, the probiotics groups showed a significantly higher abundance of these strains than in the placebo groups at the 4-week endpoint (Figure 3).

**Figure 4:**
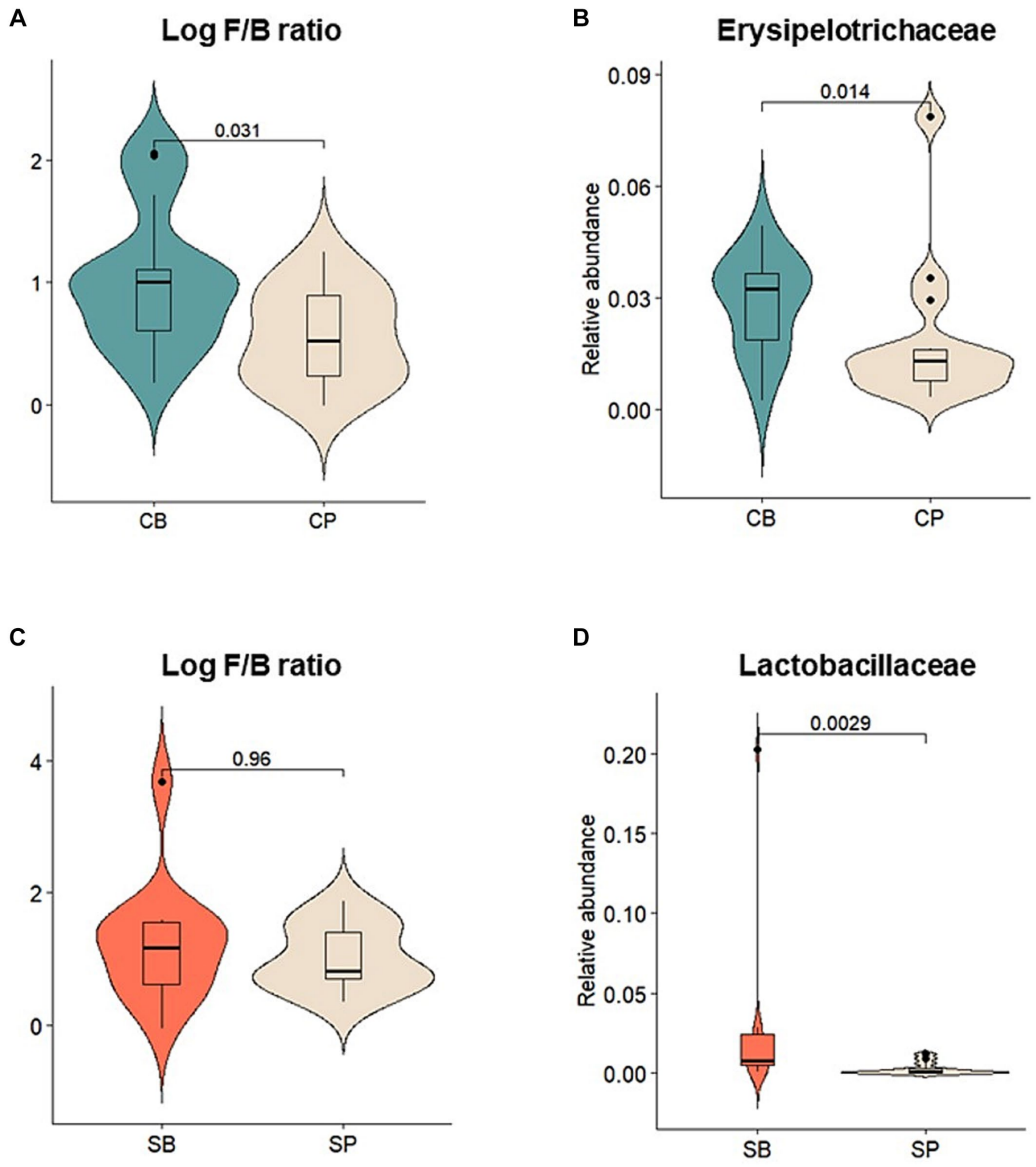
Comparison of the relative abundances of prominent microbial taxa in the gut microbiota after probiotic intervention in the Insensitive Gut and Sensitive Gut groups (CB and SB; *n* = 14 and *n* = 13, respectively) as well as their respective placebo groups (CP and SP; *n* = 14 and *n* = 13, respectively). **(A)** Log *Firmicutes*-to-*Bacteroidetes* (F/B) ratio and **(B)** relative abundance of *Erysipelotrichaceae* in the Insensitive Gut group. **(C)** Log F/B ratio and **(D)** relative abundance of *Lactobacillaceae* in the Sensitive Gut group. Wilcoxon rank-sum tests were conducted to assess significance.

Relative abundances of major gut microbiota at the phylum, family, and genus levels were assessed before and after intervention. After intervention, the CB group showed an increase in the abundance of *Actinobacteria*, *Firmicutes*, and *Verrucomicrobia* and a decrease in *Bacteroidetes* and *Proteobacteria*, whereas these changes were not observed in the CP group (Supplementary Figure S2A). The log *Firmicutes*-to-*Bacteroidetes* (F/B) ratio was also significantly higher in the CB group (*p* = 0.031) (Figure 4A). At the family level, the CB group exhibited a decreased abundance of *Acidaminococcaceae*, *Bacteroidaceae*, *Prevotellaceae*, and *Porphyromonadaceae* and increased abundance of *Coriobacteriaceae*, *Ruminococcaceae*, and *Erysipelotrichaceae*. In contrast, the CP group showed decreased *Bifidobacteriaceae* and increased *Prevotellaceae* abundances (Supplementary Figure S2B). Although no significant changes were observed before and after intervention, *Erysipelotrichaceae* abundance was significantly higher in the CB group at the 4-week endpoint (*p* = 0.014) (Figure 4B).

Similar trends were observed in the SG group, with increases in the abundance of *Actinobacteria*, *Bacteroidetes*, and *Verrucomicrobia* and decreases in that of *Firmicutes* and *Proteobacteria* at the phylum level after intervention (Supplementary Figure S2D). Additionally, after intervention, the SB group displayed higher abundances of *Actinobacteria* and *Verrucomicrobia* than those in the SP group. However, no significant changes were observed before and after intervention. At the family level, the SB group showed a decreased abundance of *Enterobacteriaceae*, *Lachnospiraceae*, *Lactobacillaceae*, and *Veillonellaceae* and increased abundance of *Bifidobacteriaceae*, *Erysipelotrichaceae*, and *Ruminococcaceae*. Conversely, the SP group showed a decreased abundance of *Bifidobacteriaceae* (Supplementary Figure S2E). At the 4-week endpoint, the SB group showed no statistically significant differences in the log *Firmicutes*-to-*Bacteroidetes* (F/B) ratio, but exhibited a significantly higher abundance of *Lactobacillaceae* compared to the SP group (*p* < 0.01) (Figures 4C,D).

**Figure 5:**
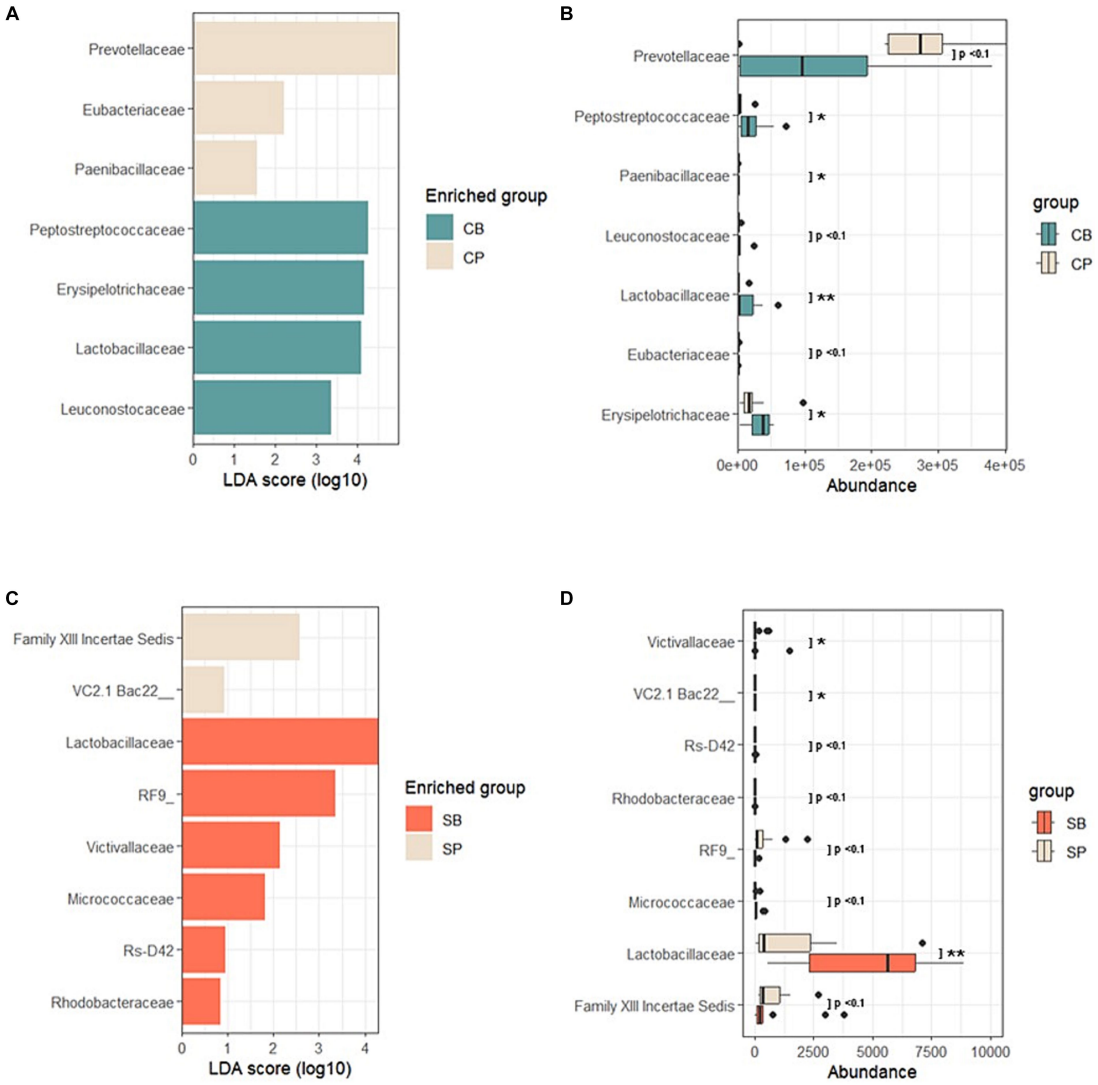
Linear discriminant analysis effect size (LEfSe) after probiotic intervention in the Insensitive Gut and Sensitive Gut groups (CB and SB; *n* = 14 and *n* = 13, respectively), as well as their respective placebo groups (CP and SP; *n* = 14 and *n* = 13, respectively). **(A)** Comparison of effect sizes between the CB and CP groups after intervention. **(B)** Abundance plot comparing the CB and CP groups. **(C)** Comparison of effect sizes between the SB and SP groups after intervention. **(D)** Abundance plot comparing the SB and SP groups. The significance level was set at *p* < 0.1 for the identified biomarkers. Significance levels are denoted in the abundance plot as follows: * (*p* < 0.05) and ** (*p* < 0.01).

Using LEfSe, a biomarker discovery tool based on linear discriminant analysis, probiotics and placebo group-specific microbial markers were identified. In the IG group, *Peptostreptococcaceae*, *Erysipelotrichaceae*, *Lactobacillaceae*, and *Leuconostocaceae* were enriched in the CB group, whereas *Prevotellaceae*, *Eubacteriaceae*, and *Paenibacillaceae* were enriched in the CP group at the 4-week endpoint (Figures 5A,B). For the SG group, *Lactobacillaceae*, *Victivallaceae*, *Micrococcaceae*, and *Rhodobacteraceae* were characteristic of the SB group, whereas the *Firmicutes*-related Family XIII Incertae Sedis and *Bacteroidetes*-related Uncultured bacterium were enriched in the SP group (Figures 5C,D).

**Figure 6:**
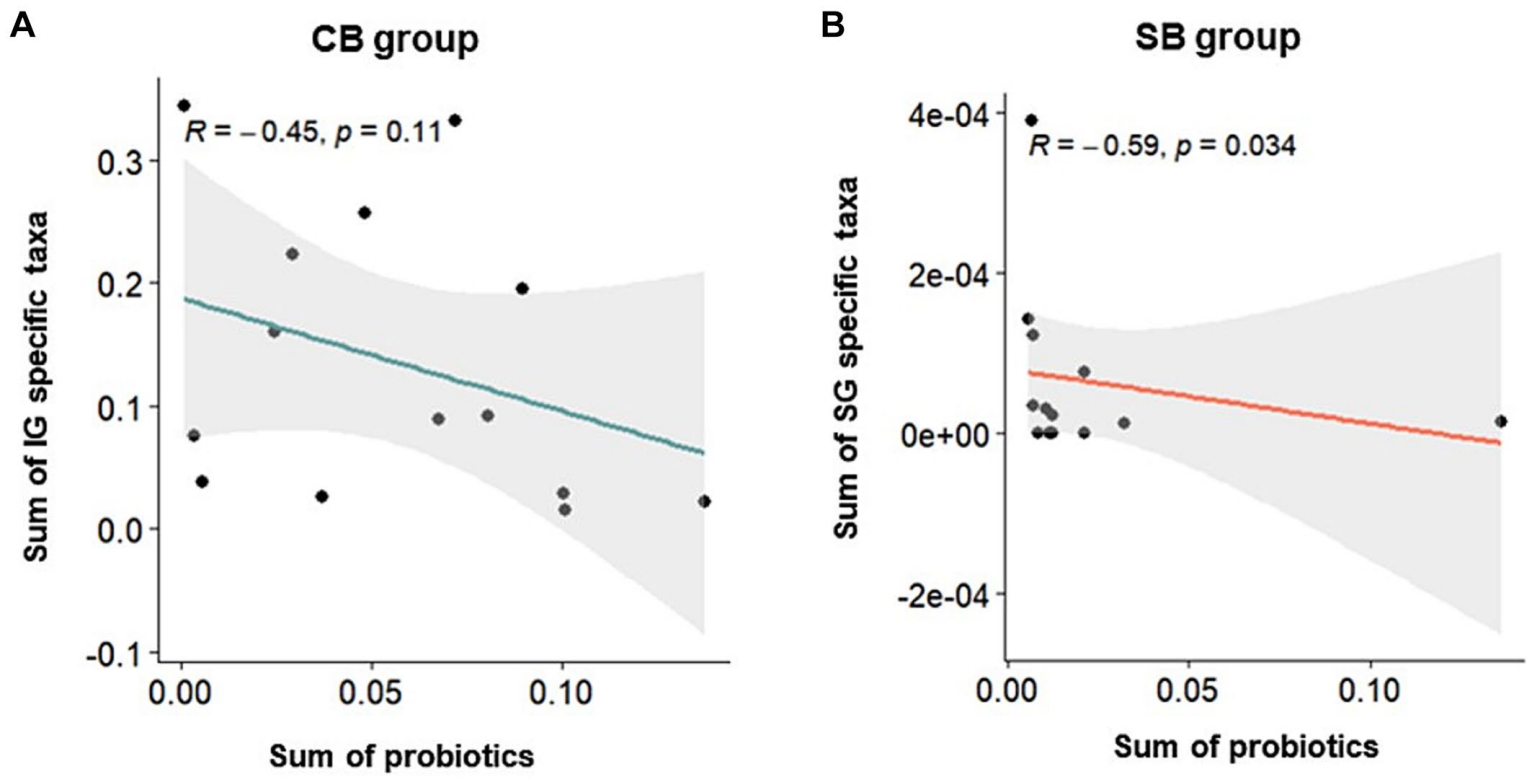
Spearman’s correlation depicting the relative abundance of probiotics and specific taxa in the Insensitive Gut (IG; *n* = 28) and Sensitive Gut (SG; *n* = 26) groups in the multi-strain probiotics intervention groups (CB and SB; *n* = 14 and *n* = 13, respectively). Scatter plots for the **(A)** CB and **(B)** SB groups after intervention.

Correlation analysis was performed using Spearman’s correlation to examine the relationship between specific characteristic microbial taxa identified through DAA and the relative abundance of intestinal probiotics after intervention in the CB and SB groups. Negative correlations were observed between characteristic indicator taxa and the relative abundance of intestinal probiotics in both the IG and SG groups (Figure 6). Notably, significant correlations were observed in the SB Group (*p* = 0.034) (Figure 6B).

## Discussion

In this clinical study, we conducted a tailored probiotics trial targeting participants with constipation- and diarrhea-dominant symptoms to analyze the separate effects of probiotic supplementation on IG and SG groups with distinct symptoms. This study assessed the efficacy of tailored probiotics by evaluating improvements in bowel habits and gut symptoms, the modulation of beneficial microorganisms, and positive compositional changes in the gut microbiome.

The multi-strain probiotic formulas provided to the participants, Consti-Biome and Sensi-Biome, have previously demonstrated efficacy in the management of specific bowel symptoms. For instance, the Consti-Biome formula, SmilinGut, exhibited efficacy in alleviating constipation symptoms in patients with constipation-predominant IBS (20). *In vitro* experiments with Consti-Biome have shown inhibitory effects against specific harmful bacteria (21), and *in vivo* studies in constipation-induced rat models demonstrated improved intestinal motility due to loperamide administration (22). Sensi-Biome includes various strains, such as *L. acidophilus* DDS-1 and *B. lactis* UABla-12, which are effective in managing abnormal stool consistency and abdominal pain in patients with IBS (23). *In vitro* inhibitory effects against specific harmful bacteria (21) and improvement in intestinal motility in an acetate-induced diarrhea rat model (unpublished data) were also observed for Sensi-Biome. This study represents the first application of Consti-Biome and Sensi-Biome probiotic formulas in humans and provides important results supporting the clinical efficacy of tailored probiotics and their ability to modulate the human gut microbiome.

In the IG group, reductions in abdominal pain, urgency and straining were observed in the probiotics (CB) group after Consti-Biome intervention. These results suggest that probiotics have a positive impact on improving constipation symptoms. Notably, the symptoms of straining are used to diagnose functional constipation under the ROME IV criteria (6). In the SG group, the probiotics (SB) group showed improved stool consistency based on the BSS after Sensi-Biome intervention. Although no significant difference was observed in incomplete evacuation, a trend towards reduction was observed in the SB group, contrasting with that in the placebo (SP) group. BSS serves as a marker for colonic transit time (24) and is a key factor in distinguishing diarrhea and constipation-predominant subtypes of IBS (25, 26).

However, the improvement in symptoms was not specific to the IG or SG groups, and the degree of improvement was mild, with some cases showing better efficacy in the placebo group, such as for the incomplete evacuation in the IG placebo (CP) group. Considering some reported clinical cases of probiotic strains found in Consti-Biome and Sensi-Biome, the relatively short 4-week intervention duration compared with the 12-week intake might explain the limited improvement of symptoms (20, 23).

Probiotic supplementation in each group was confirmed by a significant increase in specific bacterial strains. Notably, the collective abundance of specific taxa unique to the IG group, including *Succinivibrionaceae*, *Micrococcaceae*, *Porphyromonadaceae*, *Prevotellaceae*, *Peptococcaceae*, and *Alcaligenaceae*, was negatively correlated with an increase in Consti-Biome (Figure 6A). Some of these taxa, such as *Porphyromonadaceae*, *Prevotellaceae*, and *Alcaligenaceae*, have been reported to be associated with constipation symptoms in previous studies (27–29). In the SG group, distinctive taxa, including *Enterococcaceae*, *Planococcaceae*, *Enterobacteriaceae*, *Nocardiaceae*, and *Staphylococcaceae* showed a negative correlation with an increase in Sensi-Biome (Figure 6B). Among these, *Enterococcaceae*, *Enterobacteriaceae*, and *Staphylococcaceae* have been linked to diarrhea symptoms (27, 30, 31).

In the CB group, a significant increase in beneficial strains such as *Firmicutes* and *Erysipelotrichaceae*, known for short-chain fatty acid (SCFA) production, was supported by composition analysis and LEfSe results (Figures 5A,B). SCFAs produced by gut bacteria contribute to serotonin (5-HT) synthesis and secretion in enterochromaffin cells, regulating gut motility through the direct stimulation of TPH1 (32). This suggests a mechanistic explanation for the relief of constipation symptoms mediated by Consti-Biome, consistent with previous *in vivo* findings (22). Other microbial markers highlighted by LEfSe also indicated the positive effect of Consti-Biome on beneficial gut microbiota composition. The decreased abundance of *Prevotellaceae* in the CB group compared with that in the CP group may suggest an alleviation of inflammation (33), whereas the scarcity of *Eubacteriaceae*, which has been associated with reduced intestinal motility and IBS severity (34, 35), is in line with the findings in the CP group. Moreover, the increase in *Peptostreptococcaceae*, which is correlated with increased bowel movements (36), and SCFA-producing *Erysipelotrichaceae* (37) along with health-promoting lactate-producing groups, such as *Lactobacillaceae* and *Leuconostocaceae* (38), support the positive effects of Consti-Biome.

In the SB group, the microbial composition and LEfSe results reflected an increase in *Lactobacillaceae*, indicating the activation of known beneficial microbes (21–23) (Figures 5C,D). Furthermore, in addition to the Uncultured and Incertae Sedis groups, other microbial markers such as *Victivallaceae* (39), negatively correlated with abdominal cramping and pain, *Micrococcaceae* (40) contributed to mucosal barrier protection and immune response stimulation, and the rarely encountered *Rhodobacteraceae* (41, 42) in cases of diarrhea, presented characteristic microbial indicators in the SB group.

Hence, Consti-Biome and Sensi-Biome probiotic formulas have the potential to improve the symptoms of constipation and diarrhea by modulating specific indicator taxa, promoting SCFA-producing bacteria, and regulating microbial biomarkers associated with inflammation and pain. Constipation and diarrhea are distinct clinical symptoms (6), and their microbial profiles have consistently exhibited variations related to each symptom (7, 27). Despite the feasibility of tailored probiotic approaches, attempts to prescribe symptom-specific probiotics are relatively limited. This study suggests the possibility of tailored probiotic prescriptions through microbial modulation and symptom improvement and underscores the need for clinical approaches that individualize probiotics for the targeted characteristics of each study subject. These findings offer insights into understanding the role of the gut microbiota in gastrointestinal health, fostering the development of therapeutic approaches for digestive disorders through microbial manipulation. However, further research and confirmation are warranted to advance clinically applicable probiotic therapies, considering the characteristics of individual study participants: there were some limitations to this study. In this clinical trial, there were a small number of participants, which limits the generalizability of the conclusions. In addition, short-term recruitment of participants based on self-reporting of constipation and diarrhea symptoms may lead to inconsistencies in symptom presentation. It is possible that different levels of intensity and frequency of symptoms exist among study participants because these symptoms result from various factors, such as diet, lifestyle, and stress. Consequently, long-term symptoms assessments with larger sample sizes must be used in future studies in order to maintain symptom consistency.

Among the aforementioned taxa, there are instances that do not specifically align with previous research findings on bowel habits. For instance, *Prevotellaceae,* which are related to microbial features in the IG group, have been reported to be associated with enterotypes and rapid gut transit, in contrast to the existing results (16). This may be due to the influence of ethnicity, age, sex, and diet on these taxa or microbial diversity (2, 14, 43). Although they may not be distinctly segregated in terms of constipation and diarrhea, they could be linked to the inflammation associated with dysbiosis, potentially contributing to the exacerbation of gastrointestinal disorders due to weakened intestinal cell function (33).

Although this study analyzed the clinical effects of probiotic consumption tailored to two types of bowel habits, it did not consider metabolomic analysis. Recent evidence showing close associations between metabolic pathways and over 95% of fecal metabolites underscores the need to understand complex interactions between the microbiome and human metabolic environment (44). From this perspective, this study lacks a comprehensive understanding of the potential efficacy of probiotics in modulating the microbiome and metabolic pathways. Future research on probiotics should accurately assess microbial metabolic activity and contribute to a better understanding of their impact on human health.

The results presented in this study demonstrate the potential clinical applicability of tailored probiotics. It is still necessary to conduct further research to enlarge the participation pool by incorporating variables such as ethnicity, diet, and lifestyle. In addition, larger sample sizes are required for long-term studies to produce more robust and reliable results. In the future, this research could provide a better understanding of the potential benefits of probiotic prescriptions tailored to individuals with compromised intestinal function.

## Conclusion

The consumption of Consti-Biome significantly improved urgency and abdominal pain compared with the placebo group. In addition, participants who consumed Sensi-Biome showed distinct improvements in stool consistency compared with placebo groups. From a gut microbiome perspective, it was observed that, depending on the bowel habit, the probiotics either enhanced microbial biomarkers associated with bowel motility, such as *Erysipelotrichaceae*, or modulated microbial biomarkers related to inflammation mitigation, such as *Lactobacillaceae*. These findings emphasize the potential of using personalized probiotics based on bowel habits. The results of this study also underscore the need for a multidimensional approach, including long-term consumption and observation, consideration of individual variations, and metabolomics when assessing the efficacy of personalized probiotics. Further research informed by a deeper understanding of patients with bowel disorders could facilitate the employment of effective, tailored probiotic treatments.

## Data availability statement

The sequences reported in this study are deposited in the ‘Sequence Read Archive’ under the accession number PRJNA1036719.

## Ethics statement

This clinical study was reviewed and approved by the Korea National Institute for Bioethics Policy (KoNIBP) (IRB: P01-202209-06-001). The studies were conducted in accordance with the local legislation and institutional requirements. The participants provided their written informed consent to participate in this study. Written informed consent was obtained from the individual(s) for the publication of any potentially identifiable images or data included in this article.

## Author contributions

UM: Conceptualization, Data curation, Formal analysis, Investigation, Methodology, Project administration, Visualization, Writing – original draft, Writing – review & editing. Y-JJ: Conceptualization, Data curation, Formal analysis, Investigation, Methodology, Project administration, Writing – review & editing. YJ: Investigation, Project administration, Writing – review & editing. JL: Conceptualization, Supervision, Validation, Writing – review & editing. B-YK: Validation, Supervision, Writing – review & editing.

## References

[1] Knight R Callewaert C Marotz C Hyde ER Debelius JW McDonald D. The microbiome and human biology. Annu Rev Genomics Hum Genet. (2017) 18:65–86. doi: 10.1146/annurev-genom-083115-022438 28375652

[2] Human Microbiome Project Consortium. Structure, function and diversity of the healthy human microbiome. Nature. (2012) 486:207–14. doi: 10.1038/nature11234, PMID: 22699609 PMC3564958

[3] Qin J Li R Raes J Arumugam M Burgdorf KS Manichanh C. A human gut microbial gene catalogue established by metagenomic sequencing. Nature. (2010) 464:59–65. doi: 10.1038/nature08821, PMID: 20203603 PMC3779803

[4] Yano JM Yu K Donaldson GP Shastri GG Ann P Ma L. Indigenous bacteria from the gut microbiota regulate host serotonin biosynthesis. Cell. (2015) 161:264–76. doi: 10.1016/j.cell.2015.02.047, PMID: 25860609 PMC4393509

[5] Sperber AD Bangdiwala SI Drossman DA Ghoshal UC Simren M Tack J. Worldwide prevalence and burden of functional gastrointestinal disorders, results of Rome foundation global study. Gastroenterology. (2021) 160:99–114.e3. doi: 10.1053/j.gastro.2020.04.014, PMID: 32294476

[6] Lacy BE Mearin F Chang L Chey WD Lembo AJ Simren M. Bowel disorders. Gastroenterology. (2016) 150:1393–1407.e5. doi: 10.1053/j.gastro.2016.02.031 27144627

[7] Pozuelo M Panda S Santiago A Mendez S Accarino A Santos J. Reduction of butyrate- and methane-producing microorganisms in patients with irritable bowel syndrome. Sci Rep. (2015) 5:1–12. doi: 10.1038/srep12693, PMID: 26239401 PMC4523847

[8] Bielecka M. “Probiotics in food”, in: Chemical and functional properties of food components.ed. Sikorski Z. (Boca Raton, FL, USA: CRC Press) 3rd ed. (2006) 20061236:413–26.

[9] FAO/WHO. Joint FAO/WHO working group report on drafting guidelines for the evaluation of probiotics in food. Food and Agricultural Organization of the United Nations. Rome, Italy: FAO. (2002). 30 p.

[10] Wieërs G Belkhir L Enaud R Leclercq S Philippart de Foy JM Dequenne I. How probiotics affect the microbiota. Front Cell Infect Microbiol. (2020) 9:454. doi: 10.3389/fcimb.2019.00454, PMID: 32010640 PMC6974441

[11] Zmora N Zilberman-Schapira G Suez J Mor U Dori-Bachash M Bashiardes S. Personalized gut mucosal colonization resistance to empiric probiotics is associated with unique host and microbiome features. Cell. (2018) 174:1388–1405.e21. doi: 10.1016/j.cell.2018.08.041, PMID: 30193112

[12] Veiga P Suez J Derrien M Elinav E. Moving from probiotics to precision probiotics. Nat Microbiol. (2020) 5:878–80. doi: 10.1038/s41564-020-0721-1, PMID: 32393856

[13] Asnicar F Leeming ER Dimidi E Mazidi M Franks PW al Khatib H. Blue poo: impact of gut transit time on the gut microbiome using a novel marker. Gut. (2021) 70:1665–74. doi: 10.1136/gutjnl-2020-323877, PMID: 33722860 PMC8349893

[14] Gupta VK Paul S Dutta C. Geography, ethnicity or subsistence-specific variations in human microbiome composition and diversity. Front Microbiol. (2017) 8:1162. doi: 10.3389/fmicb.2017.01162, PMID: 28690602 PMC5481955

[15] Rosas-Plaza S Hernández-Terán A Navarro-Díaz M Escalante AE Morales-Espinosa R Cerritos R. Human gut microbiome across different lifestyles: from hunter-gatherers to urban populations. Front Microbiol. (2022) 13:1–10. doi: 10.3389/fmicb.2022.843170, PMID: 35558108 PMC9087276

[16] Vandeputte D Falony G Vieira-Silva S Tito RY Joossens M Raes J. Stool consistency is strongly associated with gut microbiota richness and composition, enterotypes and bacterial growth rates. Gut. (2016) 65:57–62. doi: 10.1136/gutjnl-2015-309618, PMID: 26069274 PMC4717365

[17] Walters W Hyde ER Berg-Lyons D Ackermann G Humphrey G Parada A. Improved bacterial 16S rRNA gene (V4 and V4-5) and fungal internal transcribed spacer marker gene primers for microbial community surveys. mSystems. (2016) 1:1–10. doi: 10.1128/msystems.00009-15, PMID: 27822518 PMC5069754

[18] Quast C Pruesse E Yilmaz P Gerken J Schweer T Yarza P. The SILVA ribosomal RNA gene database project: improved data processing and web-based tools. Nucleic Acids Res. (2013) 41:D590–6. doi: 10.1093/nar/gks1219, PMID: 23193283 PMC3531112

[19] R Core Team (2021) ‘R: A language and environment for statistical computing’. Vienna, Austria: R Foundation for Statistical Computing. Available at: http://www.r-project.org.

[20] Mezzasalma V Manfrini E Ferri E Sandionigi A la Ferla B Schiano I. A randomized, double-blind, placebo-controlled trial: The efficacy of multispecies probiotic supplementation in alleviating symptoms of irritable bowel syndrome associated with constipation. Biomed Res Int. (2016) 2016:1–10. doi: 10.1155/2016/4740907, PMID: 27595104 PMC4993960

[21] Jang YJ Min B Lim JH Kim BY. In vitro evaluation of probiotic properties of two novel probiotic mixtures, ConstiBiome and Sensi-biome. J Microbiol Biotechnol. (2023) 33:1149–61. doi: 10.4014/jmb.2303.03011, PMID: 37386724 PMC10580887

[22] Jeong JJ Ganesan R Jin YJ Park HJ Min BH Jeong MK. Multi-strain probiotics alleviate loperamide-induced constipation by adjusting the microbiome, serotonin, and short-chain fatty acids in rats. Front Microbiol. (2023) 14:1174968. doi: 10.3389/fmicb.2023.1174968 37333632 PMC10272585

[23] Martoni CJ Srivastava S Leyer GJ. *Lactobacillus acidophilus* DDS-1 and *bifidobacterium lactis* UABla-12 improve abdominal pain severity and symptomology in irritable bowel syndrome: randomized controlled trial. Nutrients. (2020) 12:1–15. doi: 10.3390/nu12020363, PMID: 32019158 PMC7071206

[24] Lewis SJ Heaton KW. Stool form scale as a useful guide to intestinal transit time. Scand J Gastroenterol. (1997) 32:920–4. doi: 10.3109/00365529709011203, PMID: 9299672

[25] Drossman DA Hasler WL. Rome IV—functional GI disorders: disorders of gut-brain interaction. Gastroenterology. (2016) 150:1257–61. doi: 10.1053/j.gastro.2016.03.035, PMID: 27147121

[26] Patel P Bercik P Morgan DG Bolino C Pintos-Sanchez MI Moayyedi P. Prevalence of organic disease at colonoscopy in patients with symptoms compatible with irritable bowel syndrome: cross-sectional survey. Scand J Gastroenterol. (2015) 50:816–23. doi: 10.3109/00365521.2015.1007079, PMID: 25636675

[27] Chong PP Chin VK Looi CY Wong WF Madhavan P Yong VC. The microbiome and irritable bowel syndrome—a review on the pathophysiology, current research and future therapy. Front Microbiol. (2019) 10:1–23. doi: 10.3389/fmicb.2019.01136, PMID: 31244784 PMC6579922

[28] Kwon HJ Lim JH Kang D Lim S Park SJ Kim JH. Is stool frequency associated with the richness and community composition of gut microbiota? Int. Res. (2019) 17:419–26. doi: 10.5217/ir.2018.00149, PMID: 30704159 PMC6667361

[29] Pan R Wang L Xu X Chen Y Wang H Wang G. Crosstalk between the gut microbiome and colonic motility in chronic constipation: potential mechanisms and microbiota modulation. Nutrients. (2022) 14:3704. doi: 10.3390/nu14183704, PMID: 36145079 PMC9505360

[30] Chung The H Le SNH. Dynamic of the human gut microbiome under infectious diarrhea. Curr Opin Microbiol. (2022) 66:79–85. doi: 10.1016/j.mib.2022.01.006, PMID: 35121284 PMC9758627

[31] Ohkusa T Koido S Nishikawa Y Sato N. Gut microbiota and chronic constipation: a review and update. Front Med. (2019) 6:1–9. doi: 10.3389/fmed.2019.00019, PMID: 30809523 PMC6379309

[32] Margolis KG Cryan JF Mayer EA. The microbiota-gut-brain Axis: from motility to mood. Gastroenterology. (2021) 160:1486–501. doi: 10.1053/j.gastro.2020.10.066, PMID: 33493503 PMC8634751

[33] Larsen JM. The immune response to Prevotella bacteria in chronic inflammatory disease. Immunology. (2017) 151:363–74. doi: 10.1111/imm.12760, PMID: 28542929 PMC5506432

[34] el-Salhy M Mazzawi T Hausken T Hatlebakk JG. The fecal microbiota transplantation response differs between patients with severe and moderate irritable bowel symptoms. Scand J Gastroenterol. (2022) 57:1036–45. doi: 10.1080/00365521.2022.2064725, PMID: 35486073

[35] Kim JE Choi YJ Lee SJ Gong JE Jin YJ Park SH. Laxative effects of phlorotannins derived from ecklonia cava on loperamide-induced constipation in SD rats. Molecules. (2021) 26:1–19. doi: 10.3390/molecules26237209, PMID: 34885790 PMC8659160

[36] Gan D Chen J Tang X Xiao L Martoni CJ Leyer G. Impact of a probiotic chewable tablet on stool habits and microbial profile in children with functional constipation: a randomized controlled clinical trial. Front Microbiol. (2022) 13:1–10. doi: 10.3389/fmicb.2022.985308, PMID: 36071965 PMC9441913

[37] Louis P Flint HJ. Formation of propionate and butyrate by the human colonic microbiota. Environ Microbiol. (2017) 19:29–41. doi: 10.1111/1462-2920.13589 27928878

[38] Kim J Bang J Beuchat LR Kim H Ryu JH. Controlled fermentation of kimchi using naturally occurring antimicrobial agents. Food Microbiol. (2012) 32:20–31. doi: 10.1016/j.fm.2012.05.007, PMID: 22850370

[39] Azcarate-Peril MA Ritter AJ Savaiano D Monteagudo-Mera A Anderson C Magness ST. Impact of short-chain galactooligosaccharides on the gut microbiome of lactose-intolerant individuals. Proc Natl Acad Sci USA. (2017) 114:E367–75. doi: 10.1073/pnas.1606722113, PMID: 28049818 PMC5255593

[40] Shao H Zhang C Wang C Tan Z. ‘Intestinal mucosal bacterial diversity of antibiotic-associated diarrhea (AAD) mice treated with Debaryomyces hansenii and Qiweibaizhu powder’, 3. Biotech. (2020) 10:392–11. doi: 10.1007/s13205-020-02383-2, PMID: 32832342 PMC7429618

[41] Ma X Zhang Y Xu T Qian M Yang Z Zhan X. Early-life intervention using exogenous fecal microbiota alleviates gut injury and reduce inflammation caused by weaning stress in piglets. Front Microbiol. (2021) 12:1–17. doi: 10.3389/fmicb.2021.671683, PMID: 34177852 PMC8222923

[42] Qiao B Liu J Xiao N Tan Z Peng M. Effects of sweeteners on host physiology by intestinal mucosal microbiota: example-addition sweeteners in Qiweibaizhu powder on intestinal mucosal microbiota of mice with antibiotic-associated diarrhea. Front Nutr. (2022) 9:1–13. doi: 10.3389/fnut.2022.1038364, PMID: 36337643 PMC9631320

[43] Gorvitovskaia A Holmes SP Huse SM. Interpreting prevotella and bacteroides as biomarkers of diet and lifestyle. Microbiome. (2016) 4:1–12. doi: 10.1186/s40168-016-0160-7, PMID: 27068581 PMC4828855

[44] Visconti A le Roy CI Rosa F Rossi N Martin TC Mohney RP. Interplay between the human gut microbiome and host metabolism. Nat Commun. (2019) 10:4505. doi: 10.1038/s41467-019-12476-z, PMID: 31582752 PMC6776654

