# Supplementary material for: Personalized probiotic strategy considering bowel habits: impacts on gut microbiota composition and alleviation of gastrointestinal symptoms via Consti-Biome and Sensi-Biome
Source: Front Nutr. 2024 Feb 16;11:1302093. doi: 10.3389/fnut.2024.1302093 (PMC10904615; doi:10.3389/fnut.2024.1302093)
Supplement: Supplementary file 1 [file Presentation_1.pptx]

## Slide 1
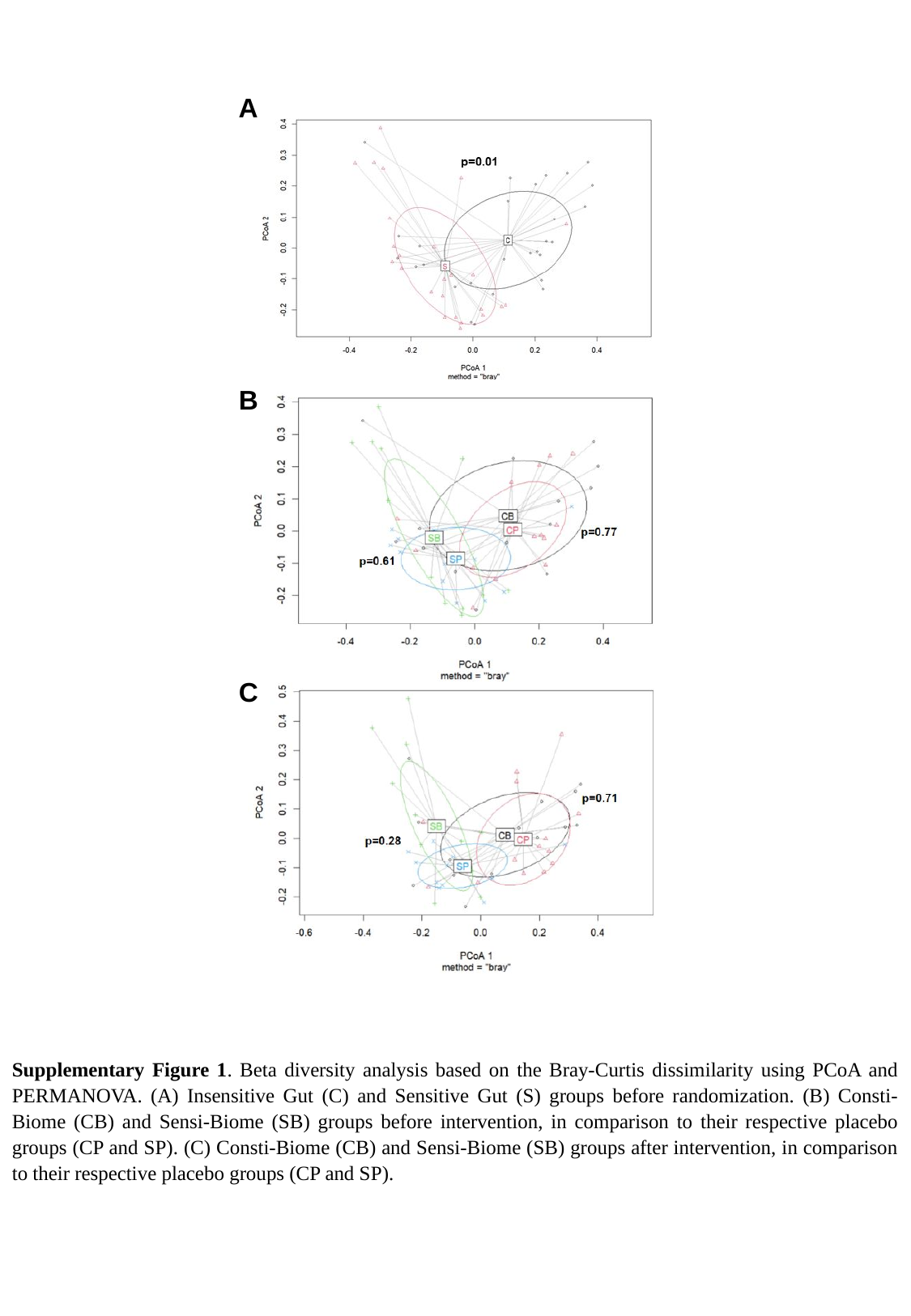

A
B
C
Supplementary Figure 1. Beta diversity analysis based on the Bray-Curtis dissimilarity using PCoA and PERMANOVA. (A) Insensitive Gut (C) and Sensitive Gut (S) groups before randomization. (B) Consti-Biome (CB) and Sensi-Biome (SB) groups before intervention, in comparison to their respective placebo groups (CP and SP). (C) Consti-Biome (CB) and Sensi-Biome (SB) groups after intervention, in comparison to their respective placebo groups (CP and SP).

## Slide 2
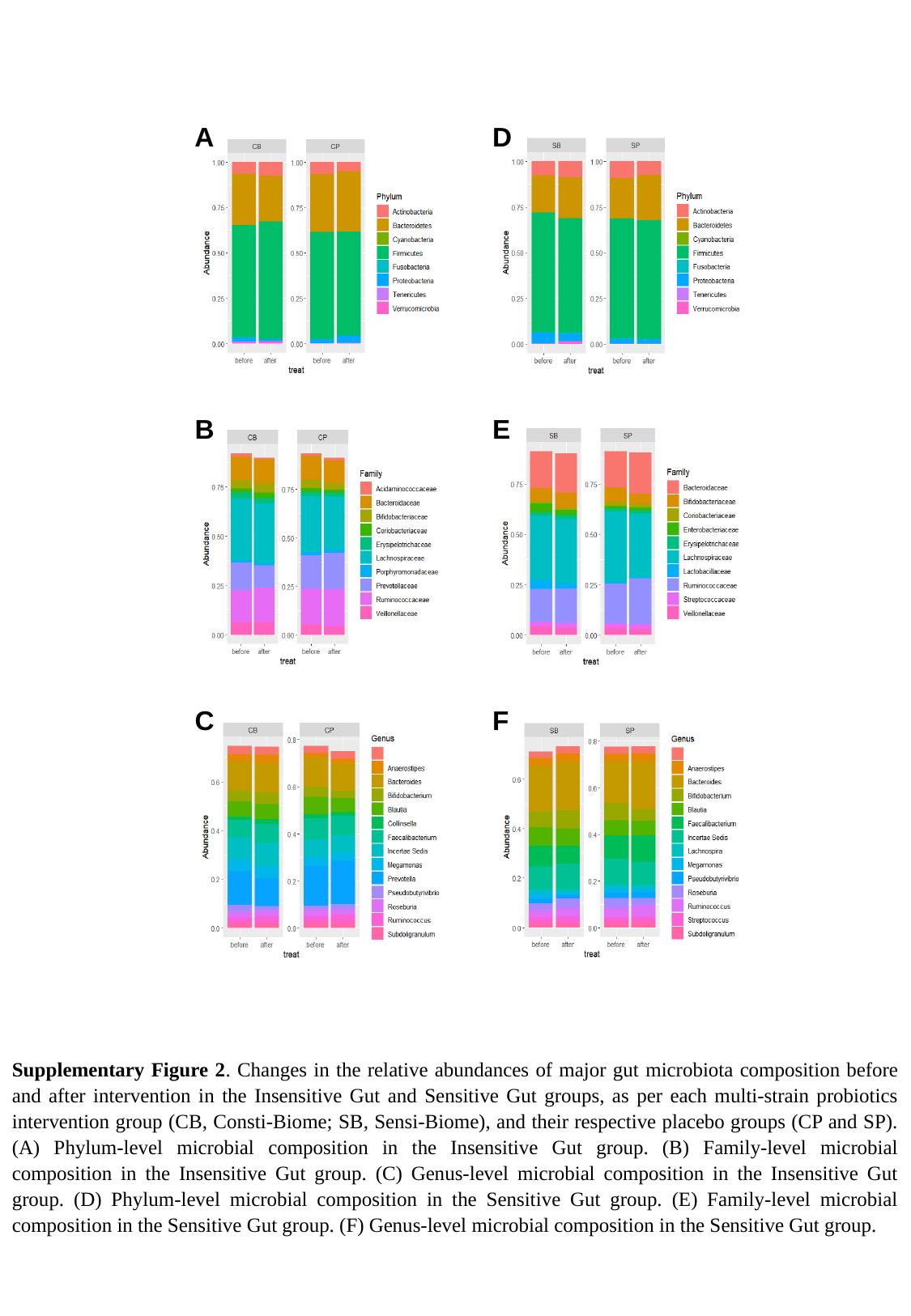

A
D
B
E
C
F
Supplementary Figure 2. Changes in the relative abundances of major gut microbiota composition before and after intervention in the Insensitive Gut and Sensitive Gut groups, as per each multi-strain probiotics intervention group (CB, Consti-Biome; SB, Sensi-Biome), and their respective placebo groups (CP and SP). (A) Phylum-level microbial composition in the Insensitive Gut group. (B) Family-level microbial composition in the Insensitive Gut group. (C) Genus-level microbial composition in the Insensitive Gut group. (D) Phylum-level microbial composition in the Sensitive Gut group. (E) Family-level microbial composition in the Sensitive Gut group. (F) Genus-level microbial composition in the Sensitive Gut group.

## Slide 3
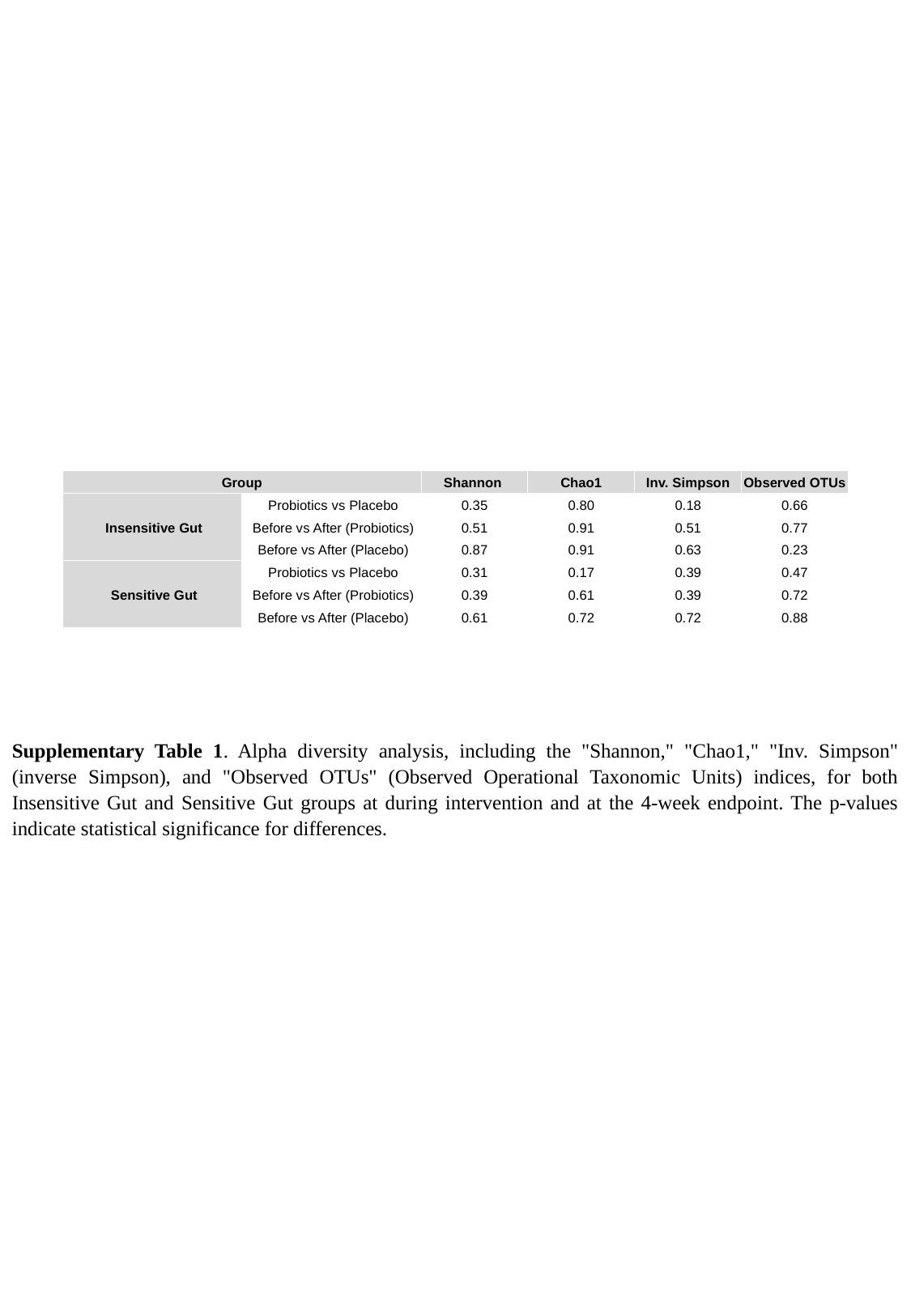

| Group | | Shannon | Chao1 | Inv. Simpson | Observed OTUs |
| --- | --- | --- | --- | --- | --- |
| Insensitive Gut | Probiotics vs Placebo | 0.35 | 0.80 | 0.18 | 0.66 |
| | Before vs After (Probiotics) | 0.51 | 0.91 | 0.51 | 0.77 |
| | Before vs After (Placebo) | 0.87 | 0.91 | 0.63 | 0.23 |
| Sensitive Gut | Probiotics vs Placebo | 0.31 | 0.17 | 0.39 | 0.47 |
| | Before vs After (Probiotics) | 0.39 | 0.61 | 0.39 | 0.72 |
| | Before vs After (Placebo) | 0.61 | 0.72 | 0.72 | 0.88 |
Supplementary Table 1. Alpha diversity analysis, including the "Shannon," "Chao1," "Inv. Simpson" (inverse Simpson), and "Observed OTUs" (Observed Operational Taxonomic Units) indices, for both Insensitive Gut and Sensitive Gut groups at during intervention and at the 4-week endpoint. The p-values indicate statistical significance for differences.
